# Biosensors to Diagnose Chagas Disease: A Brief Review

**DOI:** 10.3390/s17112629

**Published:** 2017-11-15

**Authors:** María-Isabel Rocha-Gaso, Luis-Jesús Villarreal-Gómez, Denis Beyssen, Frédéric Sarry, Marco-Antonio Reyna, Carlos-Napoleón Ibarra-Cerdeña

**Affiliations:** 1 Departamento de Ciencias Computacionales, Universidad de Quintana Roo, Unidad Cancún, 77519 Cancún, Mexico; mrocha@uqroo.edu.mx; 2 Escuela de Ciencias de la Ingeniería y Tecnología, Cuerpo Académico de Bioingeniería y Salud Ambiental, Universidad Autónoma de Baja California, 22260 Tijuana, Mexico; luis.villarreal@uabc.edu.mx (L.-J.V.-G.); investigador.reyna@gmail.com (M.-A.R.); 3 Institut Jean Lamour, 54600 Villers-lès-Nancy, France; denis.beyssen@univ-lorraine.fr (D.B.); frederic.sarry@usherbrooke.ca (F.S.); 4 Departamento de Ecología Humana, Cinvestav Unidad Mérida, 97310 Mérida, Mexico

**Keywords:** Chagas disease, biosensors, detection technologies, diagnosis

## Abstract

Chagas disease (CD), which mostly affects those living in deprived areas, has become one of Latin America’s main public health problems. Effective prevention of the disease requires early diagnosis, initiation of therapy, and regular blood monitoring of the infected individual. However, the majority of the *Trypanosoma cruzi* infections go undiagnosed because of mild symptoms, limited access to medical attention and to a high variability in the sensitivity and specificity of diagnostic tests. Consequently, more affordable and accessible detection technologies capable of providing early diagnosis and *T. cruzi* load measurements in settings where CD is most prevalent are needed to enable enhanced intervention strategies. This work analyzes the potential contribution of biosensing technologies, reviewing examples that have been tested and contrasted with traditional methods, both serological and parasitological (i.e., molecular detection by PCR), and discusses some emerging biosensing technologies that have been applied for this public health issue. Even if biosensing technologies still require further research efforts to develop portable systems, we arrive at the conclusion that biosensors could improve the accuracy of CD diagnosis and the follow-up of patients’ treatments in terms of the rapidity of results, small sample volume, high integration, ease of use, real-time and low cost detection when compared with current conventional technologies.

## 1. Introduction

Chagas disease (CD), discovered in 1909 by the Brazilian physician Carlos Chagas [1], nowadays represents one of the greatest Latin American public health concerns [2]. In terms of the disease burden estimated by disability-adjusted life years (DALYs), CD figures as one of the most important parasitic vector-borne illnesses in the region of the Americas; DALYs for CD are more than five times higher than malaria and twice higher than dengue [3], and yet it is still absent in the agenda of the public health policies and practices of many endemic countries [4,5]. Furthermore, this disease ranks fourth in mortality and eighth in morbidity among global neglected tropical diseases [6], and it is estimated that between eight and eleven million people are infected, while 100 million are at risk of acquiring the disease. CD is caused by the parasitic presence of the *Trypanosoma cruzi* (*T. cruzi*) in the organism, which is mainly transmitted by contamination with the infected feces of blood-sucking triatomine vectors during a human blood meal. Nevertheless, it can also be transmitted through blood transfusions [7], organ transplants, infected mothers to their unborn children and by the ingestion of contaminated food (i.e., oral transmission [8]). Although disease progression can be associated with the mechanism of infection, with oral transmission causing the most severe outbreaks [9], people living in regions at risk of infection are susceptible to polyparasitism (i.e., coinfections and superinfections with different strains of *T. cruzi*), with unknown effects on the variability of the disease progression and response to treatments [10].

CD mostly affects people in socioeconomically deprived areas and the majority of the *T. cruzi* infections go undiagnosed because of generally mild symptoms and limited access to medical care [11]. Due to this fact, CD is considered as a neglected tropical disease, whose improvement in diagnosis and treatment today requires research and development efforts with non-profit interests. The highest prevalence of Chagas disease has been reported in Bolivia (6.75–15.4%), followed by Paraguay (0.69–9.3%), Panama (0.01–9.02%), Brazil (0.8–1.30%), Mexico (0.5–6.8%) and Argentina (4.13–8.2%) [12]. Citing a case, this disease causes almost 6% of the annual deaths in Mexico, and the seroprevalence can roughly be estimated to be at least 3% due to a non-existent epidemiological surveillance. However, less than 0.5% of the infected individuals have access to treatment in Mexico as a result of anachronisms in the normativity or even because of misconceptions among medical professionals, alongside other failures in the public health system [13].

Although CD mainly affects Latin American countries, with the increasing ease of travel and migration, other countries are also being affected by this infection [14]. Several cases have been reported in the USA, Canada, Europe and in Western Pacific regions such as Japan and Australia [14,15,16]. Notably, CD continues to be an inconspicuous public health problem, with limited medical awareness [17]. Thus, the treatment of CD urgently requires new standardized diagnostic test procedures.

**Figure 1:**
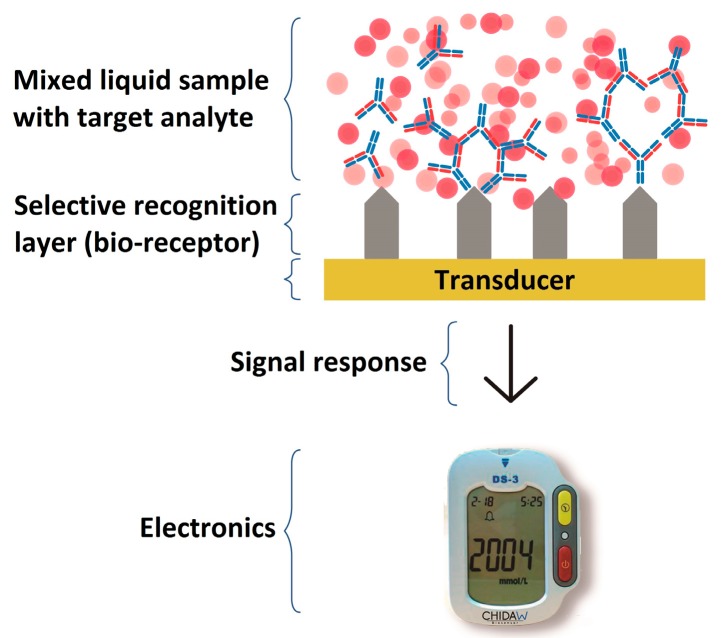
General scheme of a biosensor detection strategy. A biosensor is composed of a biochemical interface where specific bio-species are recognized; a transducer which translates the recognition event to another physical response that can be measured and an electronic system which acquires and records the signal.

Biosensors are relatively new analytical devices that can help to detect the presence of specific compounds and pathogens in liquid environments and complex mixtures such as: water and blood serum. Although these devices have been formerly used in the alimentary industry (mostly to detect toxins and infectious pathogens), they are being increasingly used to diagnose human diseases [18]. Therefore, these devices can be employed for the diagnosis of CD. The development of biosensors requires a biologically active component to be immobilized onto the surface of a transducer. The selective recognition layer towards *T. cruzi* specific antigens present in patients’ blood serum can selectively detect the target analyte, generating a signal response in the sensor (see Figure 1). Depending on the type of transducer that is employed, biosensors can be electrochemical, acoustical or optical.

In this work, we firstly introduce a brief description of the disease. Secondly, we present a review of biosensor technologies whose applicability to diagnose CD has been investigated. Finally, we mention the benefits and drawbacks of applying biosensors as solutions to this major public health issue and also explore the infrastructure required to conduct biosensor experiments for this application.

## 2. Brief Description of CD and Current Needs

CD passes through two successive stages: an acute phase and a chronic phase. The acute phase occurs during the 6–8 weeks following infection. This stage can be followed by an indefinite period (also known as the indeterminate phase) in which an equilibrium between the parasite and the immunological response of the infected individual is reached, and most infected patients appear healthy, with no evidence of organ damage that could be found by current standard methods of clinical diagnosis [8]. However, several patients progress to the chronic phase of CD, which lasts for the rest of the life of an infected individual, and has different forms. Several years after the chronic phase has started, 10–40% of infected individuals will pass to the cardiac form of the disease and will develop conditions of various organs, mainly the heart, the digestive system, and occasionally, the peripheral nervous system [8,19]. These important symptomatic changes occur 10–20 years after the vector’s contact and include a broad range of damage. Clinical manifestations vary from mild symptoms to heart failure and, frequently, sudden cardiac death [19]. Currently, the acute phase of CD is recognized only in an estimated 1–2% of all individuals acquiring the infection [8] due to a lack of access to sufficient medical care. Thus, more than any other parasitic disease, CD is closely related to social and economic development.

**Figure 2:**
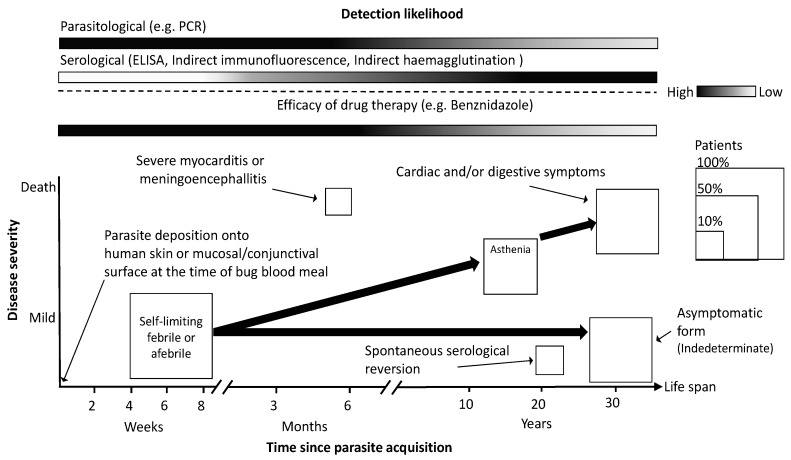
Schematic representation of Chagas disease severity evolution. Disease severity is presented along a time axis beginning with parasite acquisition via vectorial infection (see text for other forms of parasite infection). Big arrows show the condition’s progression from a state to another and the size of boxes represents the proportional frequencies of patients presenting that particular condition. Gradient bars show the likelihood of parasite detection by different test types and the drug treatment efficacy along the time axis.

Figure 2 shows a simplified schematic representation of disease progression in severity (leading to the patients’ death) along the life span of infected individuals, showing the most representative forms and clinical manifestations of CD (for a more detailed characterization, see ref. [20]). It is important to note that disease severity does not necessarily depends on the disease phase (i.e., acute, indeterminate, chronic) or time since parasite acquisition, as a relatively small proportion of patients can rapidly progress to a symptomatic form and die from myocarditis or meningoencephallitis if not treated [20]. Most of the patients who are susceptible to such fatalities are young infants or people who suffer from immunosuppression, and when transmission is oral [21]. Likewise, a moderate proportion of patients will abide in the indeterminate form without any symptoms for the rest of their lives [20]. There is evidence of some patients that have experienced a spontaneous cure (a serological reversion), although these events are rare [22]. When untreated, patients can experience severe fatigue (i.e., asthenia) during the initiation of the symptomatic phase of the disease. This produces a decrease in physical activity due to effort intolerance, leading CD to be one of the most important diseases that cause disability-adjusted life-years (DALYs) [23]. An important consideration to be made is that parasite infection can be treated with a favorable prognosis when patients are diagnosed early and treated in the acute phase, whereas treatment efficacy diminishes at the advanced level of disease progression. At the symptomatic period of cardiac or digestive forms, the treatment does not help to impede tissue damage progression [24]. Therefore, shortening the period of the diagnosis after parasite exposure is a key aspect of disease prognosis. As can be seen in Figure 2, choosing the convenient test type is an important criterion to have a better opportunity to detect the parasite presence. Serological tests will expand their likelihood applicability as they can reduce its detection threshold.

In order to face the epidemiological challenges due to the increasing complexity of interactions among the transmission routes of *T. cruzi* in endemic and non-endemic countries, access to early diagnostic and treatment seems to be the most cost-efficient ways of reducing the CD burden [5,11]. Seeing that the bug bite is quite notorious, and people can suspect that they are infected with *T. cruzi* when a *chinchoma* (name of the inflammatory injury after a bug bite) appears, they could search for medical assistance and be subjected to a blood test.

Climate change and global warming increase the risk of a rising CD burden in some regions. Climate change has a significant impact on vector-borne diseases [25] and undoubtedly detonates variables that make CD transmission more dangerous, as the WHO points out [26]. Nevertheless, the incidence of CD can be greatly reduced by residual insecticide-based vector control programs that decrease the populations of the transmitting vectors and by improving housing [27].

## 3. Current Detection Technologies and Their Limitations

Currently, laboratory methods are employed to diagnose CD, depending on the patients’ phase of infection, some methods being more convenient than others [28]. During the acute phase of CD, a large number of parasites are present in the peripheral blood and can be diagnosed by a parasitological test such as a direct microscopical observation of fresh blood and by one of several polymerase chain reaction (PCR) techniques. However, for the chronic phase of CD the parasitological and molecular diagnosis efficiency is reduced due to the scarce parasitemia. For instance, a meta-analysis of ELISA (serological) vs. PCR-based diagnosis methods concluded that the last method gave lower sensitivity (between 50% and 90%), whereas specificity was higher (100%) [29]. Therefore, the immunodiagnosis is widely used, since nearly all *T. cruzi*-infected individuals in the chronic phase develop antibodies against the complex antigenic mixture of the parasite [8].

Several immunodiagnosis tests are available, but mainly three conventional tests are widely used in the clinical diagnosis of CD: indirect haemagglutination (IHA), indirect immunofluorescence (IIF) and enzyme-linked immuno assay (ELISA). These tests present several limitations such as (i) cross-reactivity with other parasites; (ii) a lack of 100% sensitivity (sensitivity and specificity represent parameters of great clinical relevance in the evaluation of a diagnostic test. Sensitivity indicates the total absence of false-negative results, while the specificity indicates a false-positive rate [28]); (iii) the need for dedicated laboratories to conduct the tests; and (iv) the requirement of long analysis times to obtain the results. IHA test results can be obtained in about two hours, whereas IIF results can be obtained after numerous steps in two hours and ELISA takes several hours to carry out, including prior sensitization of microplates with *T. cruzi* antigens for about 12 h [6]. Furthermore, all these tests have to be performed in centralized laboratories; some of them require sophisticated equipment and skilled technicians. Moreover, these methods provides qualitative results [30] and other limitations [31]. Finally, none of these tests have a sensitivity of 100%; therefore, the WHO recommends conducting at least two conventional tests for a definitive diagnosis of *T. cruzi* infection [8].

More recently, non-conventional tests, such as rapid lateral flow (RLF) tests, have become commercially available on the market to detect *T. cruzi* infection using whole blood, serum or plasma [7]. These tests are based on different test principles: immunochromatography, particle agglutination, immunofiltration or immunodot. They provide rapid results (between 5 to 60 min reading times) without the need of electrical equipment and they require low volume samples (5 to 150 μL). However, the sensitivities and specificities of such tests are lower than those of conventional tests and they only provide qualitative or semi-quantitative results, which prevents obtaining important test information like genetic lineage of the *T. cruzi* [32] and the immunoreaction kinetics.

## 4. Biosensing Research Efforts for Chagas Diagnosis

Biosensors that have been investigated for the diagnosis of CD can be classified into electrochemical and optical. Among electrochemical sensors, amperometric [33,34,35,36] and impedimetric [37] sensors can be found, whereas in optical sensors only surface plasmon resonance (SPR) transducers [6] are reported. Biosensors could provide the benefits presented in Table 1 compared to other currently employed techniques for the diagnosis of CD.

Pumpin-Ferreira et al. reported a biosensor for the diagnosis of CD in 2005 [33]. It consisted of an amperometric immunosensor which required an electrochemical interaction and, therefore, a potentiostat–galvanostat was employed to conduct the measurements. Potentiostats are powerful equipment, but they are large and heavy for a final portable biosensing system. Hence, other electronics for readout systems should be developed for these biosensors which provide higher miniaturization and integration capabilities for portable systems. In this same year, Salinas et al. also reported an amperometric immunosensor with analysis time of at most 23 min [38]. This group obtained a higher sensitivity compared to the ELISA method.

In 2011, Pereira et al. developed an electrochemical immunosensor to detect specific IgG *T. Cruzi* antibodies present in patient’s serum samples with a detection limit of 3.07 ng/mL [30]. They achieved this result by functionalizing the sensor with *T. cruzi* proteins from epimastigote membranes as antigens and by the electrodeposition of gold nanoparticles where the *T. cruzi* antigens were immobilized. For this work, the group employed a potentiostat for the measurements. The authors stated a slight electrocatalytic effect due to the use of the gold nanoparticles which increase the sensitivity of the device and the efficiency of the *T. cruzi* antigen’s immobilization.

Recently, Luz et al. (2015) presented the first biosensor for the diagnosis of CD based on SPR transducers [6]. They obtained the parameter related to the presence of antibodies anti-*T. cruzi* found in human serum in approximately 20 min. In 2016, this same group observed that the immunoassay they conducted discriminated CD from other infectious diseases with a higher percentage of accuracy compared to ELISA and also presented a higher sensitivity—of 100%—compared to other diagnostic methods, such as PCR, which has a sensitivity of 90% and a satisfactory specificity of 97.2% [28]. These facts, in our opinion, prove the SPR technique to be very promising for CD diagnosis. Nevertheless, the use of SPR transducing principle currently leads to high volume and heavy commercial apparatus, since it requires the integration of a light source, for the laser generation and light detectors. Thus, this technology is currently only suitable for laboratory tests. Moreover, even if optical biosensors can be highly sensitive, the cost of SPR equipment is higher than USD $50,000. For this reason, not many researchers can afford such systems [39].

Corina et al. developed a portable electrochemical biosensor platform for serodiagnosis of infectious diseases based on magnetic microbeads [40]. The portability of this platform was achieved by employing a mini-portable potentiostat with eight channels designed and manufactured by the group. They successfully demonstrated the use of this platform for the diagnosis of CD with assays reading times of 20 s, in which they obtained results similar to those of ELISA in terms of sensitivity and selectivity. However, this system is not commercially available yet. In addition, the technique requires the detection of electrochemical reactions to conduct the assays, which leads to indirect steps. Other detection techniques, such as the use of acoustic sensors, could avoid these steps in the future.

In 2016, Regiart et al. also reported an electrochemical immunosensor for anti-IgM T. cruzi-antibodies [49]. They employed gold nanoparticles to improve the sensor’s limit of detection by increasing its active surface area. The achieved detection limit in this work was 3.03 ng/mL.

In this same year, Janissen et al. deployed a nanowire biosensor based on Field-effect transistor (FET) technology that achieved a limit of detection of approximately 6 fM for the CD protein marker IBMP8-1 [43], which remarks the potential of this highly sensitive biosensor for the control of this disease.

## 5. Biosensors and Their Contribution to Reducing the CD Burden

An ideal serological test should be easy to perform in a single step, fast, cheap, require no special equipment or refrigeration reagents, and should have a sensitivity and specificity of 100%. Such a test does not currently exist for the diagnosis of CD. Hence, new technologies, which combine, robustness, simplicity, portability and rapidity with an effective sensitivity and selectivity, could contribute to more efficiently diagnose CD. There is evidence to show that biosensors could meet most of these attributes for this application [6,33]. Biosensors could improve the diagnosis and the patients’ treatment follow-up in terms of rapidity, real-time and low cost detection compared to current detection technologies such as PCR and ELISA. In addition, the use of biosensors offers significant advantages such as small fluid volume manipulation, a high integration capability that facilitates the development of portable devices and ease of use. This should allow their use by non-specialized personnel in non-centralized laboratories [18]. Nevertheless, further research efforts are needed to achieve a biosensing portable device for CD diagnosis.

PCR can be employed in series or in parallel with a serological test to diagnose chronic CD. Nowadays, this technique is the only one that can provide genotype capabilities. However, the diagnosis of CD diseases with this technique has a serious drawback, due to the fact that the principle of this technique relies on the need to find the parasite in patients’ blood samples, which in the chronic phase of the disease is unlikely [50]. Therefore, PCR should not be ordered as a unique test to diagnose CD in its chronic phase neither.

The expected features of biosensors are high selectivity and sensitivity, real-time label-free monitoring, ease of use, reliability, high miniaturization capabilities and low cost. Biosensors based on optical or acoustic wave sensing technologies could meet these requirements in the near future and seem to be very promising tools for this application. Moreover, such devices will lead to more sensitive tests at lower reagent concentrations, allowing biosensing system users to (i) reduce the cost of reagents; and (ii) obtain valuable quantitative information; and (iii) extend the measurement range of the assays. Table 2 presents some biomarkers that could be detected using biosensors in each phase of CD. The detection of such biomarkers might improve the disease diagnosis and/or treatment.

## 6. Infrastructure Requirements

To develop a portable biosensor system for the rapid diagnosis of CD, it is necessary first of all to integrate a transducer with a suitably sensitive bio-chemical layer. Some authors have already achieved this milestone, as stated in Section 4. Additionally, the system requires the integration of (i) an electronic read-out system, for the interrogation and signal acquisition; (ii) a microfluidic system, to handle bio-fluids; and (iii) a thermal control unit, to keep the temperature stable during the sample analysis. This last point could be avoided if it is proven that the temperature sensitivity of the sensor in use is negligible for the experiments we are conducting or if a differential measurement setup for a compensation of temperature is employed [51]. Furthermore, it is important to mention, that a fully-automated feature is desirable for the complete system, in order to run the sample analysis as comfortable as possible.

Nowadays, some companies offer commercial solutions for integrated biosensing systems that could be employed to diagnose CD. However, most of these systems are still of considerable size, weight and price, which prevents their wide use for field applications in low income communities. Table 3 shows some integrated biosensing platforms currently available in the market and some of their features. As can be appreciated in the table, all of these systems require to be operated in a laboratory due to their dimensions and weight. If researchers choose a non-commercial solution, they are required to design and develop a whole system according to their needs. Nevertheless, this might allow them to pursue a more compact, cost-effective and portable system.

## 7. Conclusions

Today, a vaccine for CD does not exist. Therefore, vector control and diagnostic tests are the most effective methods for preventing the disease and applying effective drug treatments. Even if a prophylactic or therapeutic vaccine could be achievable in next few years, this would need to be part of integrated efforts that include better diagnostic means, since a vaccine is unlikely to be enough to stop the parasite transmission. Thus, highly predictive diagnostic tests are required, not only to estimate the real size of the CD problem, but also to assess the effectiveness of every action that is taken towards a disease burden reduction.

Currently, there are three conventional tests to diagnose Chagas in its chronic phase: IHA, IIF and ELISA. All of these tests have sensitivities under 100%. Therefore, the WHO recommends performing at least two of these tests for a conclusive diagnosis, leading to a bottleneck of parasite detection, caused by limited local availability of laboratories in which such tests can be performed. In addition, the diagnosis of the disease is generally delayed due to logistic restrictions for potential patients to access diagnostic centers.

Non-conventional qualitative tests, such as RLF tests, are currently commercially available. Some of these tests can lead to results within minutes, but they cannot be considered as conclusive tests by themselves. Biosensors could be employed to support RLF test results in the future, diminishing the overall time necessary to achieve definitive quantitative results. Moreover, biosensors could exceptionally contribute to a fast and secure screening method for blood banks in small and medium health facilities. Hence, biosensors could improve CD diagnosis and the patients’ treatment follow-up in terms of rapidity, small sample volume, high integration, ease of use, real-time, label-free and low cost detection compared with current conventional tests. Pursuing these goals is of considerable importance and interest to diminish the CD burden and to reduce the risk of the intensified spread of the disease due to climate change and ease of travel. Nevertheless, further research efforts are still needed to develop portable biosensing systems in order to effectively employ this technology for CD diagnosis nearer to those patients living in deprived areas, since current commercially available systems are not portable yet.

## Figures and Tables

**Table 1:** Methods for the diagnosis of Chagas disease (CD). * LOD: Limit of Detection. NA: Not applicable.

Methods	Drawbacks	Benefits	LOD *	Need of Labeling	References
**Selective Media**	− Microorganism need to growth fast − Long time to yield results − It needs to be conducted in a laboratory − Needs an aseptic working area − Requires highly trained personnel − Tedious procedure	+ Low cost + Easy to perform	NA	No	[41,42]
**Biosensors**	− Not commercially available for CD diagnosis − Large dimensions − It needs to be conducted in a laboratory (currently) − Further research and development is required for portable systems − High research cost	+ Label free detection + Quantitative detection + No need of an aseptic working area + Fast (real-time measurements) + Easy to perform. Not need of trained personnel + In situ simple preparation + High analytical specificity + Reduction of reagents consumption + Reduced analysis time + High sensitivity and reliability + Integration of multiple processes in a single device + Possible automation + Low fabrication cost + Possible sensor regeneration for multiple analyses	~2 nM [43]	No	[6,18,28,30,33,34,35,36,37,43]
**ELISA**	− Labeled detection − Requires highly qualified personnel − Long assay times − It needs to be conducted in a laboratory − Complex and expensive instruments − Presents cross-reactivity with other infectious agents	+ High selectivity and sensitivity + Improves the time required to yield results + It works well for samples without interfering molecules	~30 nM [43]	Yes	[41,42,44,45,46,47]
**Quantitative PCR**	− Complex and expensive instruments are required − Requires highly trained personnel − It needs to be conducted in a laboratory − Difficult to perform − Long assay times	+ High selectivity and sensitivity + Improves the time required to yield results	~10 nM [43]	Yes	[40,42,45,46,47]
**Rapid lateral flow test**	− Specificity 96.8% − High possibility of presenting false positive − Just qualitative results − The method needs a tube and a measured volume of sample	+ Fast (15–25 min) + High sensitivity of 99.5% + Low cost (less than $2 USD to the end user)	~20 nM	Yes	[7,48]

**Table 2:** Biomarkers used for the detection of Chagas disease by the phase of the disease.

Phase of the Disease	Biomarker Name	Reference
**Acute**	IL-12, IFN-gamma, TNF-alpha, nitric oxide (NO), IL-17, IL-10, CD4+ T cells	[52]
**Chronic**	Aptamer, CCL2, MAL/TIRAP, CCR5, CD15s+ Treg cells, CD27+ T cells, CD28+ T cells, CD8+ T cells, TIMP-1, IMP-2, Troponin I, TGF-β, IL-10, APOA1, Fibronectin, MMP-2, MMP-9, ANP, BNP, N-terminal pro-BNP, IFN-γ, TNF-α, IL-1β, IL-6, CKMB, miRNA-1, miRNA-133a, iRNA-133b, miRNA-208a, miRNA-208b, PIIINP, PICP, Syndecan-4, ICAM-1, Galectin-3, KMP11, HSP70, PAR2, Tgp63, Antigen 13, SAPA, Tc24	[16,53,54,55,56,57,58,59,60,61,62,63,64]

IL: Interleukine, IFN: Interferon, TNF: Tumor Necrosis Factor, CD: Cluster Differentiation, miRNA: micro ribonucleic acid, CCL: Chemokine ligand, TIRAP: Toll-interleukin 1 receptor adaptor protein, MAL: MyD88 Adapter-Like, CCR5: C-C receptor quimiocina type 5, TIMP: Tissue Inhibitor of Metalloproteinases, IMP: Inosine Monophosphate, TGF: Transforming Growth Factor, AMP: Apolipoprotein A, MMP: Matrix Metalloproteinases, ANP: Atrial Natriuretic Peptide, BNP: Brain Natriuretic Peptide, CKMB: Creatine Kinase, subunits designated M and B, APOA: Apolipoprotein A, PIIINP: Procollagen III N-Terminal Propeptide, PICP: Propeptides of Type I Procollagen, ICAM: intercellular adhesion molecules, KMP: Kinetoplastid membrane protein, HSP: Heat Shock Proteins, PAR: Protease activated receptor, Tgp: Thymocyte growth peptide, SAPA: Sphingolipid activator protein A, Tc: *T. cruzi*.

**Table 3:** Different commercial biosensing systems currently available on the market.

Product	Company Name	Transducer Technology	Dimensions (cm)	Weight (kg)	Sample Volume (µL)	Portability Rate *
Q-Sense Omega Auto	Q-sense	Acoustic	70 × 67 × 57	83	50	1
Biacore X100	General Electric	SPR	59.6 × 56.3 × 59.3	47	20–30	3
AWS A20-F20	AWsensors	Acoustic	77 × 75 × 45	60	50–1000	2
OpenPlex	Horiba	SPRi	49 × 30.4 × 48	15.6	200	4

* Portability Rate: 1 for a non-portable equipment to 5 for a portable equipment.
